# HPC-T-Assembly: a pipeline for de novo transcriptome assembly of large multi-specie datasets

**DOI:** 10.1186/s12859-025-06121-4

**Published:** 2025-04-28

**Authors:** Franco Liberati, Taiel Maximiliano Pose Marino, Paolo Bottoni, Daniele Canestrelli, Tiziana Castrignanò

**Affiliations:** 1 Department of Ecological and Biological Sciences, University of Tuscia, Viale dell’Università s.n.c., 01100 Viterbo, Italy; 2 Department of Computer Science, Sapienza University of Rome, Viale Regina Elena 295, 00166 Rome, Italy

**Keywords:** de novo transcriptome assembly, Non-model organisms, RNA-seq, High-performance computing

## Abstract

**Background:**

Recent years have seen a substantial increase in RNA-seq data production, with this technique becoming the primary approach for gene expression studies across a wide range of non-model organisms. The majority of these organisms lack a well-annotated reference genome to serve as a basis for studying differentially expressed genes (DEGs). As an alternative cost-effective protocol to using a reference genome, the assembly of RNA-seq raw reads is performed to produce what is referred to as a ‘de novo transcriptome,’ serving as a reference for subsequent DEGs’ analysis. This assembly step for conventional DEGs analysis pipelines for non-model organisms is a computationally expensive task. Furthermore, the complexity of the de novo transcriptome assembly workflows poses a challenge for researchers in implementing best-practice techniques and the most recent software versions, particularly when applied to various organisms of interest.

**Results:**

To address computational challenges in transcriptomic analyses of non-model organisms, we present HPC-T-Assembly, a tool for de novo transcriptome assembly from RNA-seq data on high-performance computing (HPC) infrastructures. It is designed for straightforward setup via a Web-oriented interface, allowing analysis configuration for several species. Once configuration data is provided, the entire parallel computing software for assembly is automatically generated and can be launched on a supercomputer with a simple command line. Intermediate and final outputs of the assembly pipeline include additional post-processing steps, such as assembly quality control, ORF prediction, and transcript count matrix construction.

**Conclusion:**

HPC-T-Assembly allows users, through a user-friendly Web-oriented interface, to configure a run for simultaneous assemblies of RNA-seq data from multiple species. The parallel pipeline, launched on HPC infrastructures, significantly reduces computational load and execution times, enabling large-scale transcriptomic and meta-transcriptomics analysis projects.

## Background

The advent of next-generation sequencing (NGS) platforms has revolutionized the field of genomics [1]. Specifically, for RNA-seq data production, these platform technologies, with their significant reduction in associated costs, have enabled the simultaneous analysis of millions of RNA molecules, allowing a deeper understanding of the transcriptome and gene expression dynamics [2, 3]. An increasing number of laboratories and research projects have contributed to the production of massive RNA-seq datasets, whose analysis requires high-performance software [4, 5] capable of processing data on non-standard computing resources. The increasing availability of RNA-seq data for organisms lacking a well-annotated reference genome has also led to the development of advanced bioinformatics pipelines for data analysis and interpretation. These pipelines involve the phase of the de novo transcriptome assembly, an essential technique for the analysis of non-model organisms, as it enables the reconstruction of the transcriptome from only RNA-seq sequencing data [6]. The production of de novo transcriptomes is a well-established practice, widely documented in the literature, and supports many projects of great interest within the scientific community [7–12]. This methodology provides valuable insights into gene expression and transcript diversity for species lacking complete genomic information. The use of automated pipelines has made the de novo assembly more accessible, allowing even more researchers with less IT expertise to perform complex analyses efficiently [13]. Such pipelines integrate various tools and steps, from data preparation to functional annotation, streamlining the entire process of transcriptome assembly and analysis. The implementation of de novo assembly pipelines on high-performance computing (HPC) systems is another crucial aspect [14–16]. Such pipelines are designed to leverage the power of distributed computing, enabling the processing of large sequencing datasets in reduced times [17, 18]. The use of HPC is particularly advantageous for the analysis of non-model organisms, where data volumes can be substantial and require significant computational resources for assembly and annotation [19–21]. Here, we introduce a new bioinformatics software, HPC-T-Assembly, which enables users to easily perform de novo transcriptome assemblies from RNA-seq data. Execution is fast and fully automated, leveraging a High-Performance Computing (HPC) infrastructure [22]. The process does not require extensive IT skills but remains flexible if users wish to configure individual steps during the execution of the assembly pipeline. The configuration and generation of the assembly workflow for HPC are conducted through a local Web interface on the user’s computer, while the parallel code execution occurs directly on the HPC cluster [23]. An additional innovative aspect is the concurrent production of several transcriptomes when starting with RNA-seq data belonging to different organisms. This article provides a detailed overview of the stages executed by HPC-T-Assembly during the de novo transcriptome assembly process, describing the workflow from data preparation and parameter configuration to resource transfer on the supercomputing platform and subsequent pipeline launch on the HPC cluster.

## Implementation

The program’s code is written in Python [24], a language of choice for big-data applications, endowed with a wealth of specialised libraries. In particular, we have used Pandas (for manipulating data structures and numerical tables); Requests (mapping the HTTP protocol to Python’s object-oriented semantics) [25]; Biopython (a collection of tools for computational biology and bioinformatics) [26]. These libraries are automatically installed by the program and remain transparent to the user. The installation of Flask, a Python micro-framework for the development of graphical interface [27], is the user’s responsibility.

HPC-T-Assembly relies on well-established and literature-validated bioinformatics third-party software for the workflow of the de novo transcriptome assembly [28].

The entire workflow, encompassing software configuration, generation, and execution on the HPC platform, is fully automated and requires no user intervention for software or library installation beyond the required installation actions for Python and Flask, thus eliminating any need for expert supervision. Upon completion of the pipeline installation, the software provides the necessary structure to organize the output for the analyzed species, creating dedicated directories for final results as well as temporary directories for intermediate computations, which are subsequently removed. Optionally, installed modules can be removed once the assembly pipeline is complete. For each input species, the process concludes by producing a FASTA file representing the transcriptome, files for Open Reading Frames (ORFs) prediction, aligned sequencing files, transcript quantification files, and reports for statistical and analytical investigations.

### Workflow engine

**Fig. 1:**
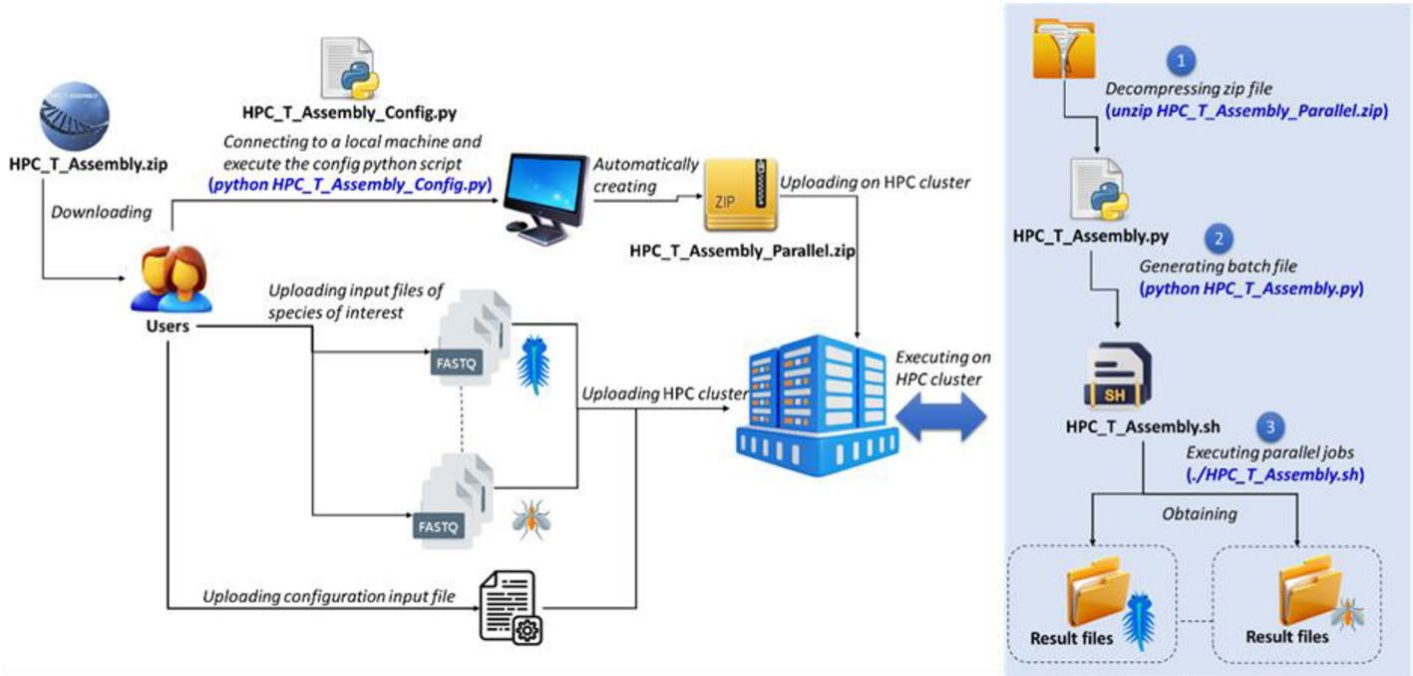
HPC-T-Assembly pipeline

Figure 1 shows an operational overview of the HPC-T-Assembly pipeline.

The diagram in Fig. 1 takes the point of view of users, describing all the steps they must perform to run the application on the HPC system. As shown, there are few steps to be completed on the local computer and a very simple procedure to be carried out on the HPC system.

Upon downloading and executing the program on a local machine, the user accesses the interface to generate an archive file that includes a launch script for the HPC platform. This archive file, along with the raw data and a text file specifying the species and dataset locations on the supercomputer’s storage system, must then be transferred to the HPC cluster. Subsequently, as shown in the blue box, the user must: (1) decompress the archive, (2) run the script to generate a batch file, and (3) either modify or directly execute the batch file. The blue-bold-italic text represents the command lines to be entered on the local machine and on the HPC system respectively.

### Interface for configuring and generating the HPC assembly pipeline

The first step is to download the program from the official website (https://github.com/poset26/HPC-T-Assembly) and save it on their local computing system. The program is distributed as an archive, which can be extracted using standard decompression tools.

The only prerequisite for the user’s system is the installation of Python (the software is implemented inPython 3, and it is recommended to use version 3.12 or later) and of the Flask microframework, both available for Windows, MAC, and Linux,and used to generate the user interface through the command ‘pip install flask’; specific hardware characteristics are not required, making the program accessible to a broad range of computing systems and portable devices (we tested our program on Android, macOS, Unix, Unix-like systems, Windows, Google Chrome OS, and Debian).

**Fig. 2:**
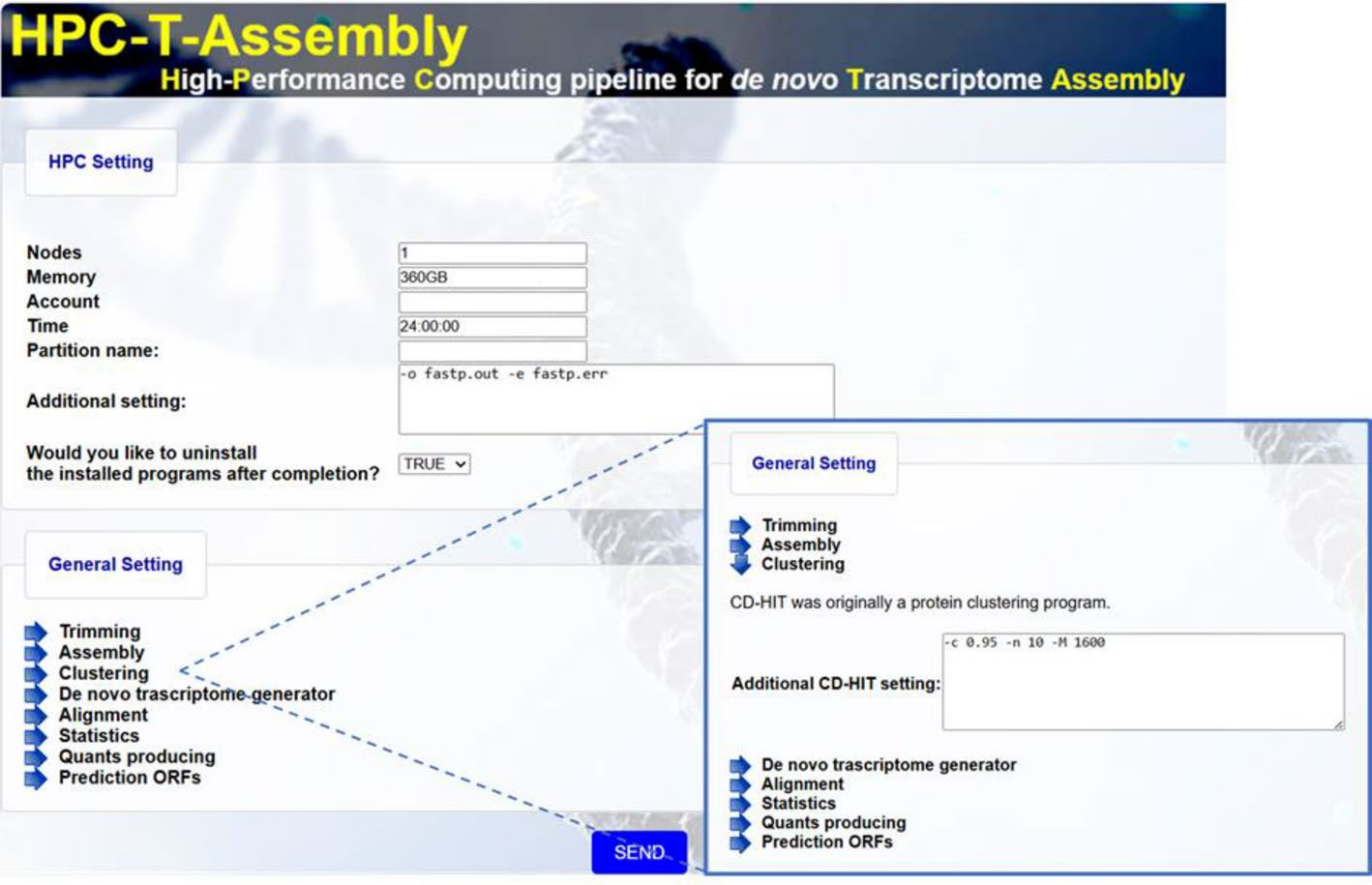
HPC-T-Assembly GUI. The upper section contains fields for setting scheduler parameters (SLURM). A drop-down menu in *General Setting* allows parameter configuration for the bioinformatics modules (by clicking on the arrow, right zoom, the user opens a legend displaying the tools in use and for each one a text-area for setting configuration parameters)

Running the configuration script (HPC_T_Assembly_Configuration.py), as described in Fig. 1, activates the Web-oriented local interface (accessible at 127.0.0.1:5000 on any browser), through which the user can set parameters for the HPC scheduling program (SLURM, Simple Linux Utility for Resource Management) and configure settings for the various third-party programs (see Fig. 2).

SLURM is an open-source workload manager and job scheduler designed for use on large-scale High-Performance Computing clusters. It is one of the most used scheduler programs in the supercomputing environment. In particular it is adopted on the supercomputers (Tier-0 and Tier-1) provided by the Center joined to The European High Performance Computing Joint Undertaking (EuroHPC JU—https://eurohpc-ju.europa.eu/). SLURM efficiently allocates computational resources, schedules jobs, and manages queues across thousands of nodes, enabling optimal utilization of HPC infrastructures. SLURM features are scalability (managing thousands of jobs simultaneously across extensive computational resources); flexibility (job prioritization, dependency handling, advanced resource allocation policies, and support for heterogeneous workloads); open-source and customizable.

This interface eliminates the need for remote server access and facilitates the use of HPC-T-Assembly for users with limited IT skills, increasing the accessibility and wider use of the proposed program. The only task required of the user is to transfer the files to the HPC infrastructure and then execute the commands (using interface tools such as PowerShell or SSH for connections) to run HPC_T_Assembly.py, followed by either HPC_T_Assembly_Single.sh for single-organism analysis or HPC_T_Assembly_Multiple.sh for the processing of multiple organisms.

After configuring parameters and clicking on the “Send” button, the GUI generates several files (compressed in a zip archive) for the parallel computation on the HPC platform. This, along with the FASTQ files and a text file (HPC_T_Assembly_Data.txt) containing the data paths, must be transferred to the HPC workspace using a standard protocol (e.g., FTP, SFTP, HTTP) or tools cross-platform FTP, FTPS and SFTP client with intuitive graphical user interface (e.g., Filezilla, PuTTY). The text file format (see Table 1) indicates the organism name preceded by the symbol #, followed by the absolute path of the FASTQ files (single or paired-end reads, with forward/left and reverse/right reads distinguished by a comma). If several species are listed, program execution generates their respective transcriptomes.

**Table 1:** Example of data format for the configuration file *HPC_T_Assembly.txt* in the case of multiple organisms.

**#Ankistrodesmus sp** *User_path*/SRR21282135_1.fastq, *User_path*/SRR21282135_2.fastq *User_path*/SRR21282136_1.fastq, *User_path*/SRR21282136_2.fastq *User_path*/SRR21282137_1.fastq, *User_path*/SRR21282137_2.fastq **#Tetraedron minutum** *User_path*/SRR1174749_1.fastq, *User_path*/SRR1174749_2.fastq *User_path*/SRR3478626_1.fastq, *User_path*/SRR3478626_2.fastq *User_path*/SRR3478627_1.fastq, *User_path*/SRR3478627_2.fastq **#Tetraselmis chuii** *User_path*/SRR1296875_1.fastq, *User_path*/SRR1296875_2.fastq	

The data have already been published and are available on NCBI (see Availability of data and materials in the Declaration section)

### Running the assembly pipeline on cluster machine

As shown in Fig. 1, once the ZIP, FASTQ, and text files are transferred to the remote HPC system (using a FTP/SFTP protocol), the user must: (1) decompress the archive, and (2) execute the start script. The first command, after logging into the HPC system, is: *unzip HPC_T_Assembly_Parallel.zip* [command (1) in light blue box of Fig. 1] and, after the decompression, the second command is *python HPC_T_Assembly.py* [command (2) in light blue box of Fig. 1]. The output of step two is a batch file: HPC_T_Assembly_Single.sh for single-organism transcriptome assembly, or HPC_T_Assembly_Multiple.sh for assembling transcriptomes of multiple species. In the third step [command (3) in light blue box of Fig. 1], the user can choose to activate the batch file immediately on the HPC cluster (the SLURM command line is: *sbatch HPC_T_Assembly_Single.sh* or *sbatch HPC_T_Assembly_Multiple.sh*), or schedule it for deferred execution. The deferred execution option enables advanced users to review and modify the bioinformatics module commands before submission, and optionally launch the pipeline at a later time. Upon execution, the batch file automatically installs, manages, and runs the necessary pipeline modules to generate the full transcriptomes on the HPC platform, utilizing all allocated computational resources.

After completion, the parallel execution of the pipeline generates a FASTA file representing the transcriptome/s (in FASTA format), ORF prediction files (in FASTA format—with extensions pep and cds—and GFF3 format), aligned sequencing files (in BAM/SAM format), transcript quantification files (in SF format, a Salmon’s output quantification file format), and reports for statistical and analytical analysis (in TSV, TXT format). The following section provides a detailed description of the pre-processing, execution, and post-analysis stages.

### Data processing steps in the HPC-T-Assembly pipeline

**Fig. 3:**
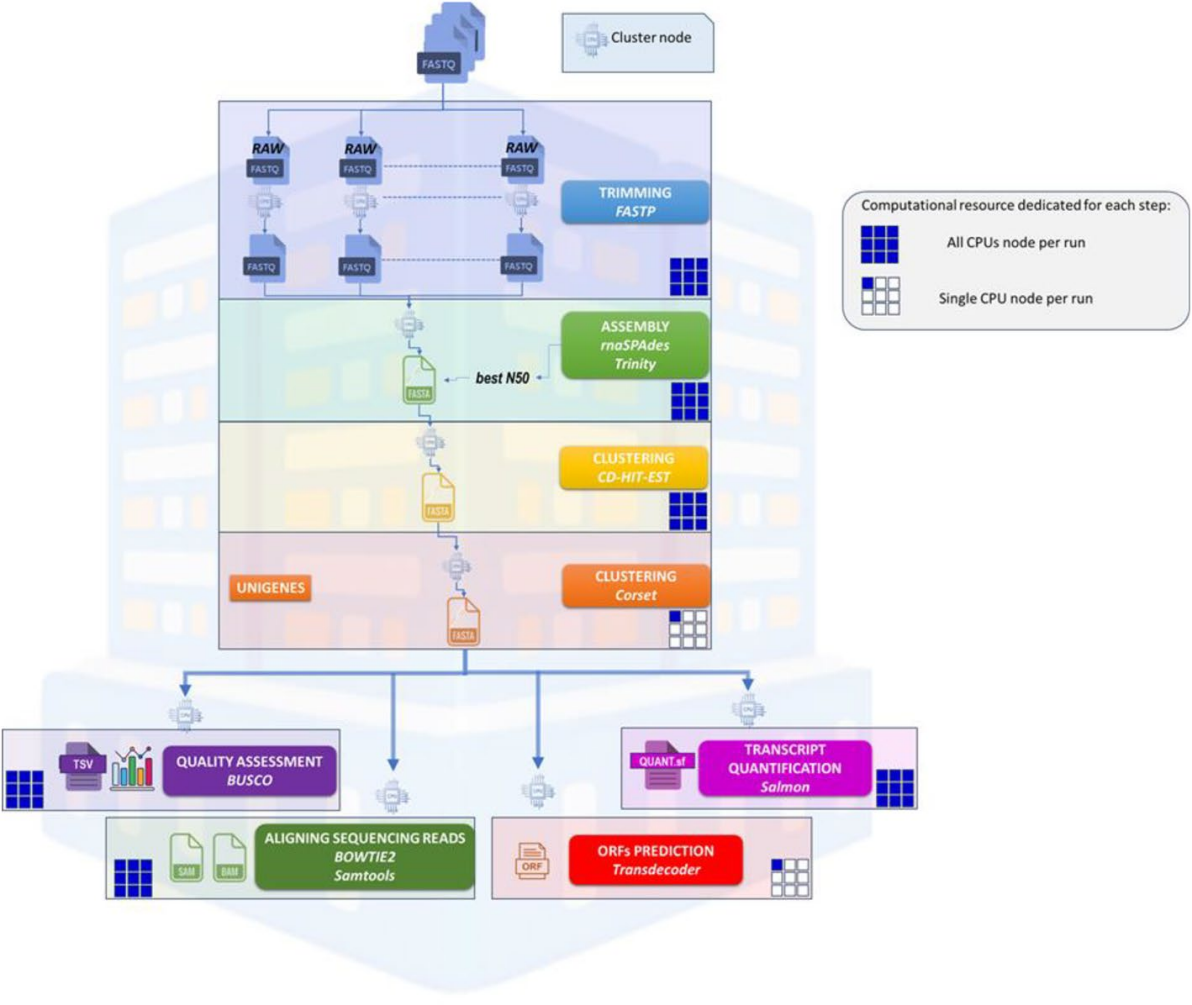
The HPC-T-Assembly pipeline. The pipeline involves three sequential stages and four parallel ones. Sequentially, the process begins with quality control and pre-assembly filtering (trimming) of the raw reads (FASTQ files). This is followed by the de novo assembly stage, where trimmed reads are gathered to both reconstruct full transcripts from nucleotide sequences and generate a FASTA file of the transcriptome. The final two sequential steps apply redundancy reduction and assembly thinning, which minimize redundant or low-informative sequences, optimizing the data for precise downstream analysis (clustering). Parallel stages are then applied to the final FASTA file (*unigenes*) and include: assembly quality assessment, alignment of the raw data to unigenes, transcript quantification, and Open Reading Frames prediction

HPC-T-Assembly constructs a de novo transcriptome from RNA-Seq data following several stages, illustrated in Fig. 3. Some of these steps require sequential processing, while others can run in parallel. Trimming is performed using sample level parallelism (see the first step, blue box, in Fig. 3), while sequential assembly and the first clustering step utilize thread-level parallelism (see the second and third steps, respectively green and yellow boxes, in Fig. 3). The second clustering step is executed sequentially due to its inherent dependencies and computational requirements (see the fourth step, orange box, in Fig. 3). Program-level parallelism is applied during quality assessment, transcript quantification, alignment of sequencing reads, and ORF prediction. CPU-per-node parallelism (single or multithreading execution) is highlighted in the legend of Fig. 3.

The process starts with a quality check and cleaning of raw reads. Trimming is performed to remove adapter sequences from the ends of reads, preventing issues during alignment and assembly. Reads with low-quality scores (based on the Phred metric in FASTQ format) are also discarded to minimize noise in subsequent analyses. HPC-T-Assembly uses the versatile FASTP (v0.23.4) [29] module for trimming large sequencing datasets quickly and efficiently, producing JSONfiles for customized analysis, removing “N” nucleotides (unidentified bases), and correcting read length discrepancies for uniform output.

For organisms without a reference genome, the protocol of the de novo transcriptome assembly reconstructs transcript sequences directly from RNA-seq reads. Assembly algorithms typically use *k*-mers, substrings of length *k*, as atomic units for reconstruction. The process, using de Bruijn graphs, builds dictionaries of all possible *k*-mers from reads. HPC-T-Assembly employs RNA-SPAdes (version 4.0.0) [30] to tackle the complexity of resolving multiple isoforms and splice-derived variants.

The assembly of RNA-Seq data, as mentioned, may propagate noise, resulting in redundant transcripts, intronic sequences, undesirable transcriptional products, and erroneous merging of similar transcripts into chimeric contigs. HPC-T-Assembly includes CD-HIT (v.4.8.1) [31] to reduce redundancy among similar transcripts, providing a highly efficient tool for managing assembly redundancy while maintaining necessary biological information.

de novo transcriptome assemblers tend to generate numerous sequences, often exceeding the number of genome-encoded genes due to transcriptional noise and alternative splicing. To streamline assembly and minimize redundant or low-informative sequences, HPC-T-Assembly utilizes Corset (v.1.09) [32] for transcript thinning and clustering refinement.

The final step conducted by HPC-T-Assembly is the parallel execution of assembly quality assessment, alignment measurement, transcript quantification, and open reading frames prediction.

Quality assessment is realized using Benchmarking Universal Single-Copy Orthologs, BUSCO, (v 1.0.0) [33, 34]. It operates by identifying sets of highly conserved, single-copy orthologous genes across different species, which are expected to be present in nearly all complete genomes of a particular taxonomic group. By examining these conserved genes, BUSCO provides an estimate of how complete a given genome assembly is, indicating the likelihood that essential genes are missing, duplicated, or fragmented within the data.

The alignment of raw and trimmed data provides a measure of how much genomic material was used to produce the final assembly. Read alignment throughBowtie2 (v.2.5.4) [35] allows the assessment of the quality of the Corset output (unigens). In order to work with compressed (binary) sequence alignment files (BAM format), widely used for processing, analyzing, and measuring sequence alignments generated by high-throughput sequencing, Samtools (v.1.21) is used [36].

The TransDecoder (v.5.7.1) [37] module is used to detect ORFs, assessing length and probability criteria based on sequence attributes and similarity to known protein domains, to predict coding genes accurately. The resulting ORF sequence files are stored in Protein Export Pathway format (PEP), CoDing Sequence format (CDS), and General Feature format version 3 (GFF3) extensions, useful for representing genomic features such as genes, exons, introns, coding sequences, and other biological characteristics. The predicted ORF sequences can be used as input for further downstream analysis of annotating de novo transcriptomes both by homology and functionally, by mapping the ORFs to databases. Usually, NR [38], SwissProt [39] and TrEMBL [40] databases are used for homology annotation whereas PFAM [39], EggNOG [41] and InterProScan [42] databases for functional annotation.

Quantification of transcripts is obtained by applying Salmon (v.1.10.1) [43], a highly efficient and accurate software for in RNA sequencing (RNA-seq) experiments. It uses a method called quasi-mapping, which does not require a full alignment of reads to the transcriptome, thereby significantly reducing computational time. Salmon builds a lightweight hash-based index of the transcriptome, allowing it to rapidly assign reads to transcript targets and estimate transcript abundances. The transcript quantification files serve as the initial input data for further downstream analysis of differential gene expression [44, 45].

**Fig. 4:**
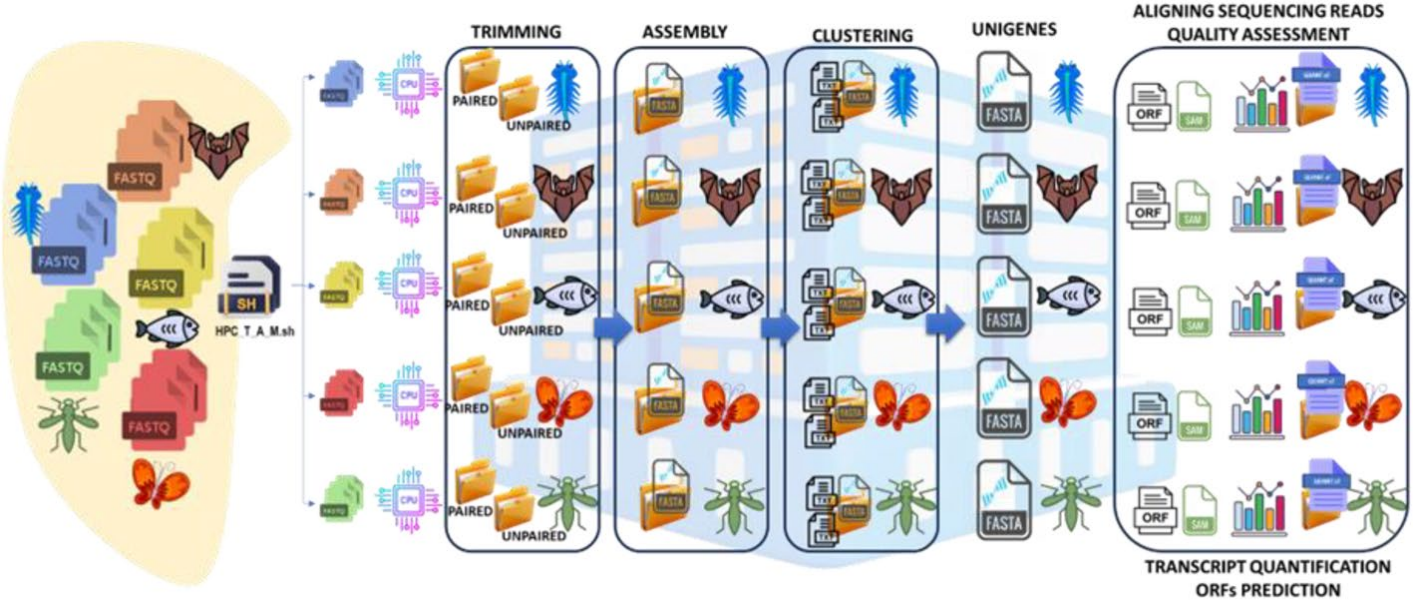
The HPC-T-Assembly parallel stages. Parallelization of pipeline steps for de novo transcriptome assembly production from different RNA-Seq datasets: the batch file identifies the raw reads for each organism and distributes them across multiple processors/cluster-nodes

As mentioned, not all stages of the assembly process can be executed concurrently (e.g., clustering). However, adopting an HPC architecture enables parallelization of transcriptome production across various organisms: all datasets are distributed among cluster-nodes and processed simultaneously, with only minimal latency due to active process queues typical of multi-user systems (Fig. 4).

The process is accompanied by correct module installation, execution and digital logs documenting system information (log files present in the main output directory). HPC-T-Assembly automates a complex workflow, from cleaning raw data, de novo transcriptome generation, clustering refinement, quality assembly assessment and measure, ORFs prediction, and transcript quantification. This reduces RNA-Seq big data processing time while requiring minimal computational expertise to utilize supercomputers effectively.

HPC-T-Assembly was tested on an HPC system with the following single-node configuration: two Intel Cascade Lake 8260 processors, each featuring 24 cores and operating at a frequency of 2.4 GHz. The node is equipped with 384 GB of memory and a minimum storage capacity of 480 GB.

Finally, HPC-T-Assembly is capable of handling errors during pipeline execution by identifying steps that have already been successfully completed and resuming processing from tasks that were either not executed or failed. To manage fault tolerance, the GUI includes a field (set to 0 by default) that triggers an error signal in case of an unwanted interruption. If the value is set by the user between 1 and 3, the system will automatically attempt to restart the process from one to three times after the error signal is triggered. As a result, the pipeline can continue without restarting from the beginning, significantly reducing execution time and improving the overall process reliability. Error analysis and recovery from the last consistent state are transparent to the user, who can obtain information about the outcome and any anomalies by reviewing the *.*log* (for generic information) and *.*verify* (summarizes the reasons for the interruption) files.

## Comparison with other tools

We compared our HPC tool with other de novo transcriptome assembly HPC pipeline tools. The comparison considered the following criteria: the presence of a graphical user interface (GUI) for parameter configuration, the need for advanced installation of parallel frameworks, the necessity of setting up virtual environments, the ability to process multi-species datasets, and scalability in HPC environments (Table 2).

**Table 2:** Comparison between HPC pipelines for de novo transcriptome assembly: usability and technical features

Tools	GUI for setting parameters	HPC support	Virtual environments	Handling multiple-species datasets	Scalability HPC
transXpress [46]	Not present	Snakemake	Conda	No	Yes
RNAflow [47]	Not present	Nextflow	Conda and docker/singularity	No	Yes
Pincho [19]	Not present	SLURM	Not needed	No	Yes
HPC-T-Assembly	Present (Windows, MacOS, Android, Unix-like)	SLURM	Not needed	Yes	Yes

Regarding processing times for multiple datasets, we demonstrate that utilizing a single node with 48 threads per dataset (for the two datasets listed in Table 1) produces the results presented in Table 3. The timing data for the clustering steps, CD-HIT-EST and Coset, correspond to the outputs generated using RNAspades, as this assembler yielded the highest N50.

**Table 3:** Benchmark of two datasets (refer to Table 1) using 1 node and 48 threads per resource on the HPC cluster Galileo100

Organism	Sample	Fastp (time)	Assembly RNA-spades (time)	Assembly trinity (time)	Clustering CD-HIT-EST (time)	Clustering corset (time)
Ankistrodesmus sp	SRR21282135	0m49.618s	80m48.9s	833m44.1s	1m4.2s	0m16.6s + 0m10.2s
	SRR21282136	0m43.176s				
	SRR21282137	0m53.720s				
Tetraedron minutum	SRR1174749	0m29.739s	256m26.8s	1041m22.4s	4m17.5s	0m13.2s + 0m9.5s
	SRR3478626	0m32.581s				
	SRR3478627	0m24.551s				

HPC-T-Assembly provides a simple, intuitive, and user-friendly graphical interface, which is not offered by the other tools analyzed. This GUI was tested on multiple operating systems (Windows, macOS, Android, and Unix-like), enabling software configuration from both mobile devices and traditional computers. The inclusion of a GUI streamlines software configuration, generation, deployment and execution on HPC platforms for non-expert users. This is a unique feature of our tool, in contrast to the other three, which rely solely on command-line interfaces. We emphasize that our tool allows non-HPC users to utilize supercomputers without needing knowledge of the scheduler or dependency syntax. Like Pincho, our tool relies on SLURM, a workload manager typically pre-installed by system administrators on HPC platforms, thereby eliminating the need for users to carry out additional installations. Two of the compared tools require installation within virtual environments (e.g., Conda, Docker, etc.), whereas neither our tool nor Pincho have such requirements. This eliminates the need for additional software installations or complex setup procedures, ensuring a fully automated and straightforward installation process. An additional and unique feature of HPC-T-Assembly is its ability to operate in parallel on multiple datasets from different species, a capability not supported by any of the other tools. This is achieved in a simple and intuitive manner by writing a text file listing the RNA-seq sample datasets for multiple species (see the example in Table 1) and uploading the file through the GUI. The generated software ensures parallel execution for each species listed in the text file. This feature, in particular, ensures the applicability of HPC-T-Assembly to large-scale data projects for studying and conservation of biodiversity. Scalability on HPC is supported by all the compared software, as they were specifically selected to operate on supercomputers.

## Conclusion

Transcriptomics is a crucial field for understanding the molecular mechanisms that underlie complex biological phenomena. This research area examines the complete set of messenger RNAs within a cell, from which proteins essential for the survival of living organisms are derived. The utilization of High-Performance Computing platforms plays a strategic role in enabling the rapid and fully automated production of de novo transcriptomes based only on RNA sequencing data.

A rapid and reliable de novo transcriptome assembly parallel workflow provides researchers with the necessary data resources for downstream analyses, including differential gene expression, annotations, inferring regulatory elements, and evolutionary patterns. This capability offers valuable insights for a wide range of studies in biomedicine, ecology, and evolutionary biology.

In this context, HPC-T-Assembly is an essential software tool for analyzing, categorizing and managing big-data information derived from RNA-Seq data. This tool significantly reduces execution times in the production of the de novo transcriptome assemblies, facilitating studies not only for a single organism but also for multiple organisms concurrently, thereby benefiting researchers in both biological and medical fields. With HPC-T-Assembly, researchers—including those without expertise in parallel computing—can work within an HPC environment simply by filling the form in a user-friendly graphical interface. Additionally, they can configure the parameters of the employed tools to meet specific requirements, perform comparative studies, or carry out temporal analyses. The module execution for de novo transcriptome assembly production requires only a bash script input from the command line, and once the FASTA file of unigenes is generated, it is immediately available for further analysis.

### Availability and requirements

Project name: HPC-T-Assembly.

Project home page: {https://github.com/poset26/HPC-T-Assembly}.

Operating system(s): Platform independent.

Programming language: Python.

Other requirements: SLURM 22.* or higher (optional) for HPC system.

License: MIT.

Any restrictions to use by non-academics: License needed.

All raw data used in this project are publicly available in the National Center for Biotechnology Information Sequence Read Archive (BIOPROJECT: PRJNA873248, PRJNA237822, and PRJNA248394).

## Data Availability

All raw data used in this project are publicly available in the National Center for Biotechnology Information Sequence Read Archive (BIOPROJECT: PRJNA873248, PRJNA237822, and PRJNA248394). HPC-T-Assembly is located at https://github.com/poset26/HPC-T-Assembly.
